# MDSCs are induced after experimental blunt chest trauma and subsequently alter antigen-specific T cell responses

**DOI:** 10.1038/s41598-017-13019-6

**Published:** 2017-10-09

**Authors:** Yvonne Hüsecken, Sylvia Muche, Monika Kustermann, Malena Klingspor, Annette Palmer, Sonja Braumüller, Markus Huber-Lang, Klaus-Michael Debatin, Gudrun Strauss

**Affiliations:** 1 Department of Pediatrics and Adolescent Medicine, University Medical Center Ulm, Ulm, Germany; 2 Institute of Clinical and Experimental Trauma Immunology, University Medical Center Ulm, Ulm, Germany

## Abstract

Severe blunt chest trauma (TxT) induces a strong inflammatory response with posttraumatic immune suppression pointing to an impaired adaptive immune response. Since CD11b^+^Gr-1^+^-expressing myeloid-derived suppressor cells (MDSCs) are induced after inflammation and suppress T cell responses, MDSC induction and their impact on T cell functions was analysed in an experimental TxT model. MDSCs were induced preferentially in the lung until 24 hours after TxT. Although MDSC numbers were only faintly increased in the spleen, splenic MDSCs isolated after TxT strongly inhibited alloantigen-induced T cell proliferation *in vitro*. Suppressive activity correlated with increased expression of arginase-1 and iNOS. MDSCs also prevented antigen-induced T cell expansion *in vivo*, since staphylococcus enterotoxin B (SEB)-induced proliferation of vβ8^+^ T cells was impaired in TxT mice in the presence of CD11b^+^Gr-1^+^ cells. Surprisingly, MDSCs were not involved in shifting T cells into Th2 cells, characterized by the secretion of cytokines impairing cell-mediated immunity and promoting immunosuppression. Instead, the presence of CD11b^+^Gr-1^+^ cells was required for efficient IL-2, IFN-γ and TNFα production after antigenic stimulation, indicating, that elevation of MDSCs early after traumatic injuries might contribute to restrict the initial inflammatory response by alleviating T cell expansion, however, without impeding Th1 functions.

## Introduction

Blunt chest trauma (TxT) has a strong impact on the morbidity and mortality of patients regardless whether it is combined with other injuries or occurred in an isolated manner^1,2^. TxT triggers a local immune response, followed by a systemic inflammation causing posttraumatic immunosuppression characterized by an impaired adaptive immune response and an increased infection risk^3–5^. Since TxT is a common but immunologically still poorly understood injury, it is of major interest to identify early changes in immune responses, which might account for a deregulated adaptive immunity.

Myeloid-derived suppressor cells (MDSCs) are cells of the innate immune system representing a heterogeneous population of immature myeloid cells, which strongly modulate the adaptive immune response. MDSCs co-express Gr-1 and CD11b. The two subsets of MDSCs are characterized by the different expression of Ly-6C and Ly-6G; granulocytic MDSCs are Ly-6G^high^Ly-6C^low^, whereas monocytic MDSCs are Ly-6G^neg^Ly-6C^high^ ^6^. MDSCs are induced and activated in the presence of various cytokines such as G-CSF, GM-CSF, IL-6, IFN-γ, IL-1β, IL-13^7^. Activated MDSCs utilize a number of mechanisms to suppress T cell functions. Immunosuppression is preferentially mediated by increased expression of arginase-1 and iNOS. Both enzymes deplete L-arginine from the microenvironment, an amino acid essentially required for T cell proliferation. ROS production, the secretion of immunosuppressive cytokines such as IL-10 and TGF-β, the induction of regulatory T cells or inhibition of T cell homing to lymphoid organs further contribute to an impaired T cell response in the presence of MDSCs^8,9^. MDSCs, however, do not only interfere with T cell activation and expansion but also modulate the type of the T cell response induced and thereby the cytokine environment. In the setting of bone marrow transplantation, viral infections, sepsis, pregnancy or in patients with certain cancers the presence of MDSCs is associated with the induction of type 2 T cells, which are characterized by the expression of Th2-specific cytokines such as IL-4, −5, −10, and −13^10–15^. Though, in models of airway inflammation MDSCs shift the balance towards Th1 cells^16,17^.

Experimental TxT leads to a local and systemic inflammatory response. As early as two hours after trauma induction pro-inflammatory cytokines and chemokines such as IL-1β, IL-6, G-CSF and MCP-1 are elevated in the BAL fluids, while the increase in plasma levels of IL-6 and G-CSF is more pronounced after 6 hours^18^. 24 hours after TxT, however, cytokine levels in plasma and BAL drop^19^. The transient increase in inflammatory mediators impacts the ability of splenic lymphocytes and macrophages to produce cytokines indicating that lung contusion causes a systemic dysfunction of immunocompetent cells located distant from the site of injury^20^.

Since cytokines such as IL-1β, IL-6 and G-CSF are released after TxT and are potent inducers of MDSCs, lung contusion might lead to MDSC accumulation and subsequent impairment of adaptive immunity. While the involvement of MDSCs after TxT is largely unknown, experimental models of sepsis, spinal cord injury or peripheral tissue trauma indicate a trauma-specific accumulation of MDSCs^21–24^. Irrespective of the model used, trauma-induced MDSCs inhibit the proliferative capacity of T cells^24–26^.

Although, severe trauma of the chest with pulmonary contusion still leads to approximately a quarter of all civil trauma-related deaths^27^, immunological alterations causing posttraumatic immune suppression are not sufficiently elucidated. Therefore, we used an established experimental, isolated TxT to clarify first whether MDSCs are induced after traumatic lung injury and secondly whether they modulate the early pro-inflammatory response and the adaptive immune response.

## Results

### Blunt chest trauma leads to the induction of MDSCs

**Figure 1:**
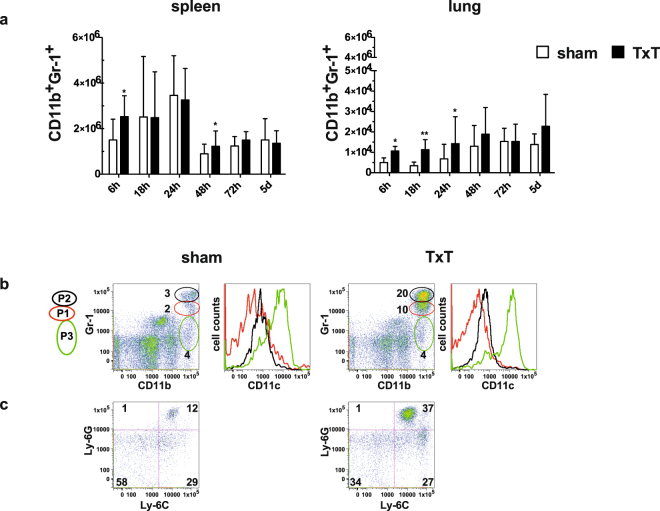
MDSCs are induced after TxT. Sham- or TxT-treated mice were analysed at different time points after TxT for the induction of MDSCs by staining splenocytes and lung leukocytes with Gr-1 and CD11b. (**a**) Absolute numbers of MDSCs were calculated in the spleen and in the lung. Data present the mean value ± SD for the following numbers of mice analysed: 6 h:n = 11; 18 h:n = 8; 24 h:n = 11; 48 h:n = 11; 72 h:n = 15; 5d:n = 15; *P ≤ 0.05; **P ≤ 0.01. Significance was calculated by Student’s t test comparing sham and TxT mice at each time point. (**b**) Mice received TxT or were sham-treated and 24 hours later lung cells were stained for CD11b, Gr-1, CD11c. Cells were gated by Gr-1 expression levels (P1 (red), P2 (black), P3 (green)). Representative flow data of one mouse out of three mice analysed are depicted and numbers represent the percentage of cells in P1, P2 and P3. Cells in P1, P2, P3 were analysed for CD11c expression. (**c**) Isolated lung cells of sham- and TxT-treated animals were stained for CD11b, CD11c, Ly-6G and Ly-6C and CD11b^+^CD11c^-^ cells were analysed for the expression of Ly-6C and Ly-6G. Representative flow data of one mouse out of three mice analysed are shown.

To clarify whether MDSCs are induced after blunt chest trauma, spleen and lungs of sham- and TxT-treated animals were analysed at different time points after trauma induction and absolute numbers of MDSCs were determined by staining cells for CD11b and Gr-1 (Fig. 1a). A slight increase of MDSCs in the spleen was observed early after 6 hours and after 48 hours compared to sham treated-animals, while at all other time points no differences were detected. However, MDSC numbers in the lungs were significantly increased in TxT-animals up to 24 hours after trauma with a peak at 18 hours. 48 hours after TxT no differences compared to sham-treated mice were detected. Surprisingly, an increase in lung MDSCs in sham-treated animals over time was observed suggesting that anesthesia and analgesic treatment might influence MDSC induction. MDSCs were identified by the co-expression of CD11b and Gr-1, both markers, which are also expressed on neutrophils. To distinguish neutrophils from granulocytic MDSCs, we subdivided the CD11b^+^Gr-1^+^ population in the lung in 3 CD11b^+^ subpopulations differing in the expression intensity of Gr-1 as described by Greifenberg *et al*.^28^. By this subdivision we obtained three populations P1–P3, which were defined by Greifenberg *et al*., as non-immunosuppressive neutrophils (CD11b^high^Gr-1^high^, P2) with a fragmented cell nuclei, as immunosuppressive granulocytic MDSCs (CD11b^high^Gr-1^intermediate^, P1) with a ring-shaped nuclei and as eosinophils and immunosuppressive monocytic cells (CD11b^high^Gr-1^low^, P3). By using this gating strategy, we observed that 24 hours after TxT neutrophils (P2) and granulocytic MDSCs (P1) were induced, while eosinophils and monocytic cells (P3) were not increased in the lung. Cells in P1 and P2 did not express CD11c, while cells in P3 express CD11c (Fig. 1b). In the CD11b^+^CD11c^−^ population, a strong increase of Ly-6G^high^Ly-6C^low^ cells consisting of neutrophils and granulocytic MDSCs was observed, while the percentage of Ly-6G^neg^Ly-6C^high^ cell representing monocytic MDSCs was unchanged (Fig. 1c). In summary, TxT induced CD11b^+^Gr-1^+^ cells, which consist of granulocytic MDSCs and neutrophils and the induction of these cells occurred early after injury and preferentially at the local site of trauma.

### Depletion of Gr-1^+^ cells does not substantially alter the early pro-inflammatory local response but interferes with the systemic pro-inflammatory response

**Figure 2:**
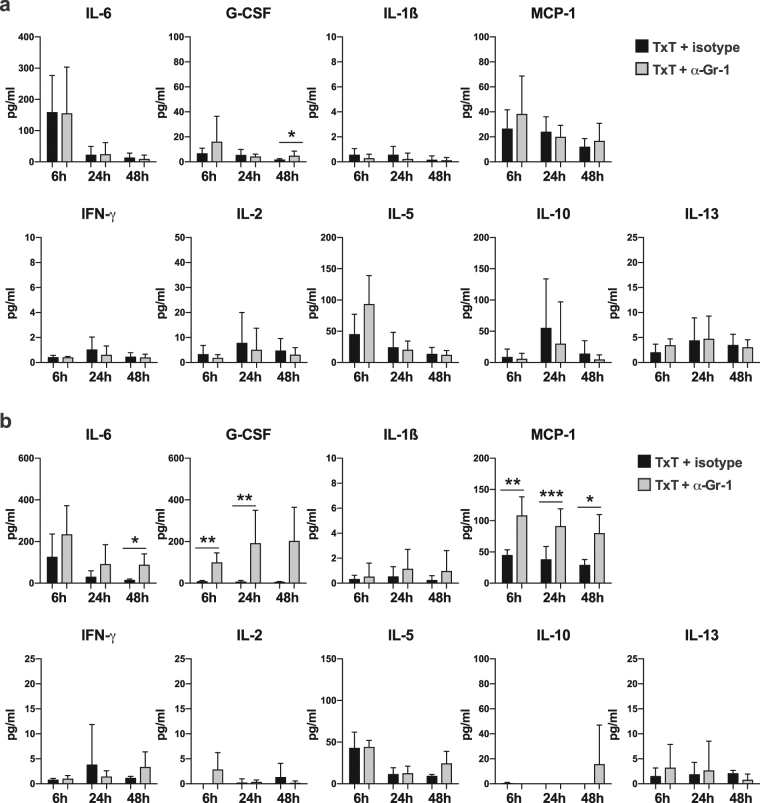
Depletion of CD11b^+^Gr-1^+^ cells does not substantially influence the expression of pro-inflammatory factors in BAL fluid but modulates the expression in the serum. TxT mice were injected with 250 µg anti-Gr-1 antibody or 250 µg isotype-specific antibody. (**a**) BAL fluid and (**b**) serum were analysed for IL-6, G-CSF, IL-1ß, MCP-1, IFN-γ, IL-2, -5, -10 and -13 concentrations at 6, 24 and 48 h after TxT. Data present the mean value ± SD for the following numbers of mice analysed: BAL:6 h:n = 6; 24 h:n = 7; 48 h:n = 8 (TxT + Isotype), n = 6 (TxT + α-Gr-1); serum: 6 h:n = 4; 24 h:n = 10 (TxT + Isotype), n = 8 (TxT + α-Gr-1); 48 h:n = 4; *P ≤ 0.05; **P ≤ 0.01; ***P ≤ 0.001. Significance was calculated by Student’s t test comparing TxT + isotype with TxT + α-Gr-1 at each time point.

To analyse the impact of Gr-1^+^ cells on the early pro-inflammatory immune response, Gr-1 expressing cells were depleted *in vivo* by anti-Gr-1 antibody treatment before TxT. Isotype specific antibody served as a treatment control. 6, 24 or 48 hours after TxT pro-inflammatory mediators and cytokines were quantified in serum and bronchoalveolar lavage (BAL) fluid. The absence of Gr-1 cells does not influence the cytokine composition in BAL fluid with the exception of a slight increase of G-CSF at 48 hours after Txt (Fig. 2a). Interestingly, in the serum of Gr-1-depleted animals MCP-1 was elevated at all time points, while G-CSF was increased at 6 and 24 hours after TxT. IL-6 concentrations in Gr-1-depleted animals were always above isotype-treated animals, however, only significantly increased at 48 hours (Fig. 2b). Concentrations of IL-1β, IFN-γ, IL-2, -5, -10 and -13 were similar in anti-Gr-1- and isotype-treated animals (Fig. 2a,b). TNFα was not detectable in BAL fluid and serum. Depleting efficiency was confirmed by flow cytometry (Supplementary Fig. S1) showing that Gr-1^high^ cells including MDSCs and neutrophils are efficiently depleted while Gr-1^low^ monocytic cells are hardly reduced. These results indicate that the presence of Gr-1^high^ cells does not influence the early local immune response but modulates the early systemic inflammatory response by decreasing pro-inflammatory factors IL-6, G-CSF and MCP-1.

### Blunt chest trauma-induced MDSCs prevent allogeneic T cell proliferation ***in vitro***

**Figure 3:**
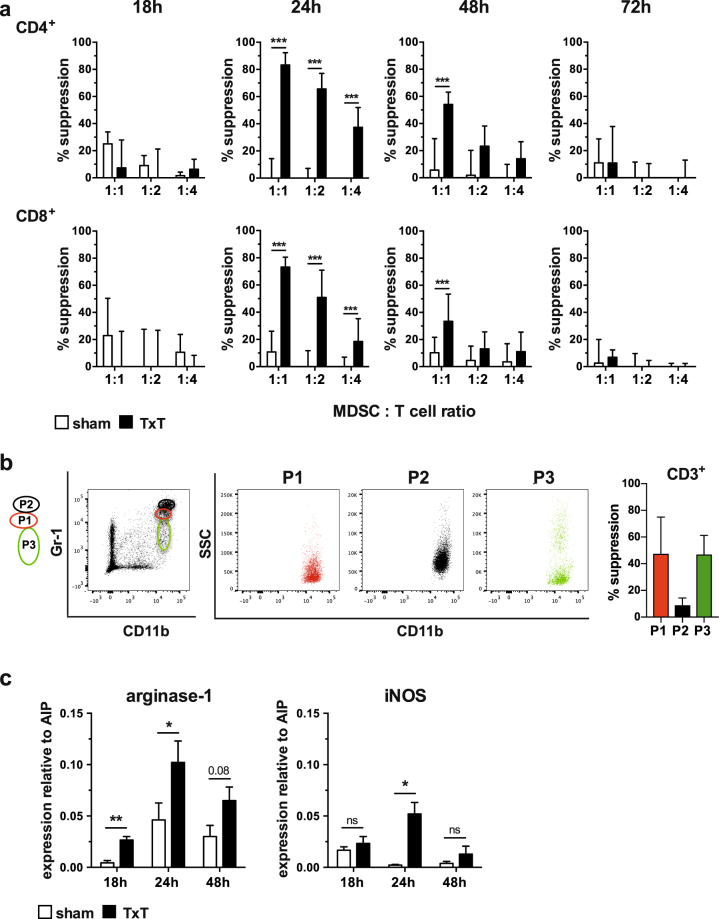
TxT-induced MDSCs are immunosuppressive. (**a**) MDSCs were isolated by magnetic beads from spleens at 18, 24, 48 and 72 hours from sham- or TxT-treated mice and co-cultivated with CFSE-labelled spleen cells of B6.SJL mice (H-2^b^, CD45.1^+^), which were activated alloantigen-specifically with irradiated spleen cells of DBA/2 mice (H-2^d^, CD45.2^+^). MDSCs were added with various cell numbers to obtain different MDSC:T cell ratios. After 4 days, cells were stained for CD45.1, CD4, and CD8 and 7-AAD. Proliferation of 7-AAD^−^ CD45.1^+^ CD4^+^ and 7-AAD^−^ CD45.1^+^CD8^+^ T cells was analysed by flow cytometry and suppression of proliferation was calculated. (**b**) Spleen cells of 6 mice receiving TxT were pooled 24 hours after TxT and subsequently B cells were depleted. Remaining cells were stained for CD11b and Gr-1. CD11b^+^Gr-1^+^ cells were sorted according to Gr-1 expression into P1, P2 and P3. Single sorted populations were depicted as SSC/CD11b. Sorted cells were co-incubated with CFSE-labelled B6.SJL cells, which were activated with allogeneic DBA/2 spleen cells at a ratio to T cells of 1:1. After 4 days, cells were stained for CD45.1, CD3 and 7-AAD and proliferation of 7-AAD^−^ CD45.1^+^ CD3^+^ T cells was analysed by flow cytometry measuring CFSE dilution and % suppression was calculated. (**c**) Splenic MDSCs were isolated from sham- or TxT-treated mice after 18, 24 and 48 hours. qRT-PCRs for the expression of the immunosuppressive molecules arginase-1 and iNOS were performed and relative expression to AIP was calculated. (**a**,**c**) Data represent the mean value ± SD of triplicates from 2 experiments (18 h, 72 h) and from 3 experiments (24 h, 48 h) with spleen cells from at least 5 mice pooled. *P ≤ 0.05; **P ≤ 0.01; ***P ≤ 0.001; ns = not significant. Significance was calculated by Student’s t test by comparing sham and TxT MDSCs at each time point and each MDSC: T cell ratio.

Although MDSC numbers did not strikingly increase in the spleen of TxT-treated mice, trauma-induced pro-inflammatory insults might influence their activation and immunosuppressive capacity. Therefore, CD11b^+^Gr-1^+^ cells including MDSCs were isolated from sham- and TxT-treated animals at different time points after trauma induction and tested for their immunosuppressiveness in mixed lymphocyte reactions (MLRs) by adding increasing numbers of MDSCs to B6.SJL-derived splenocytes (CD45.1^+^, H-2^b^) activated with irradiated DBA/2 (CD45.2^+^, H-2^d^) splenocytes. Notably, CD11b^+^Gr-1^+^ cells isolated 24 hours after trauma induction from TxT-treated mice efficiently prevented CD4^+^ and CD8^+^ T cell proliferation at all MDSC: T cell ratios tested, while cells derived from sham-treated mice did not exhibit inhibition of T cell proliferation. Immunosuppressive capacity was still detectable after 48 hours, although only at a MDSC:T cell ratio of 1:1, while T cell proliferation was unaffected in the presence of MDSCs isolated 18 or 72 hours after trauma induction (Fig. 3a). Representative flow data are depicted in Supplementary Figure S2. Since isolated CD11b^+^Gr-1^+^ cells comprises neutrophils (P2), granulocytic MDSCs (P1) and monocytic MDSCs (P3), we sorted the different subpopulations, analysed their CD11b/SSC profile and subsequently tested their immunosuppressive capacity in MLR. Neutrophils (P2) do not suppress alloantigen-specific T cell proliferation, while granulocytic (P1) and monocytic (P3) MDSCs are immunosuppressive (Fig. 3b) clearly showing that Gr-1^+^ cells consist of granulocytic and monocytic MDSCs, which inhibit T cell proliferation, while Gr-1^+^ neutrophils are not immunosuppressive. Inhibition of T cell proliferation is known to be mediated preferentially by the L-arginine metabolizing enzymes arginase-1 and iNOS. In accordance with the observed inhibition of T cell proliferation at 24 and 48 hours after trauma induction, arginase-1 and iNOS were up regulated in CD11b^+^Gr-1^+^ cells isolated from TxT-treated mice compared to CD11b^+^Gr-1^+^ cells derived from sham mice (Fig. 3c).

### Blunt chest trauma-induced MDSCs inhibit the proliferative capacity of T cells ***in vivo***

**Figure 4:**
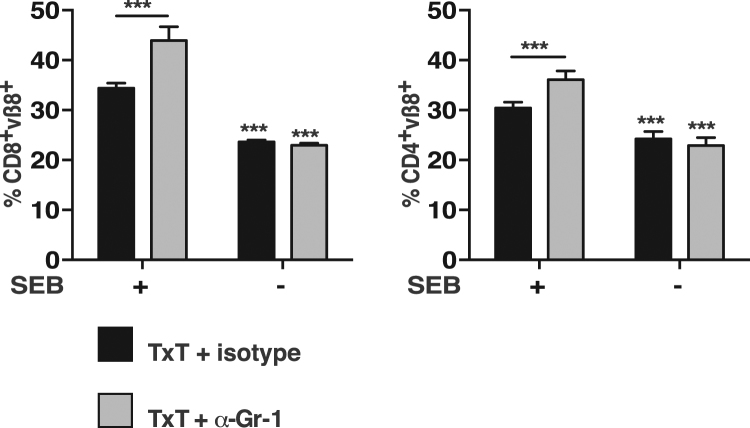
T cells from TxT-mice exhibit increased proliferation in the absence of CD11b^+^Gr-1^+^ cells after SEB injection. Mice were treated with the Gr-1-depleting antibody (α-Gr-1) or an isotype-specific antibody (isotype). TxT was induced after 24 h and after additional 24 h SEB was injected i.v. 48 h after SEB injection, splenocytes were stained for vβ8, CD4 and CD8 and the percentage of vβ8^+^CD4^+^ and vβ8^+^CD8^+^ T cells was determined by flow cytometry. Data represent the mean value ± SD of 5–8 mice/group analysed. Significance was calculated by one way ANOVA with Sidak as post test.

Since TxT-derived MDSCs exhibited an increased immunosuppressive capacity compared to sham-derived MDSCs, we determined, whether the presence of TxT-MDSCs also impairs the expansion of T cells after antigenic activation *in vivo*. *In vivo* injections of staphylococcal enterotoxins are known to activate certain T cell subsets at first and subsequently lead to anergy induction and clonal deletion at later time points^29^. Staphylococcus enterotoxin B (SEB) specifically activates T cells bearing Vβ8 TCRs and expansion of Vβ8^+^ T cells can be detected in the spleen preferentially in the CD8^+^ T cell compartment^30^. To define the influence of MDSCs on Vβ8^+^ T cell expansion, mice were either injected with the MDSC-depleting anti-Gr-1 antibody or isotype specific antibody (isotype) 24 hours before TxT induction. 24 hours after TxT, SEB was injected and Vβ8^+^ T cell expansion was determined 48 hours later. Independent of the presence or absence of Gr-1^+^ cells, about 23% of the splenic T cells express the Vβ8^+^ TCRs in the CD4^+^ and CD8^+^ T cell population. In the presence of Gr-1^+^ cells (TxT + isotype) SEB induced a slight expansion of Vß8 T cells in the CD4^+^ (-SEB:24%; + SEB:30%) and a stronger expansion in the CD8^+^ T cell population (−SEB:24%; +SEB:34%). However, if Gr-1^+^ cells including neutrophils and granulocytic MDSCs were absent (TxT + α-Gr-1), SEB injection increased the percentage of Vβ8^+^ expressing T cells from 23% to 36% in the CD4^+^ T cells and from 23% to 44% in the CD8^+^ T cells (Fig. 4). These data clearly indicate, that TxT-induced Gr-1^+^ cells including immunosuppressive MDSCs impair the proliferative capacity of antigen-stimulated T cells *in vitro* and *in vivo* early after traumatic injuries.

### Blunt chest trauma does not modulate T cell numbers in lung and spleen

**Figure 5:**
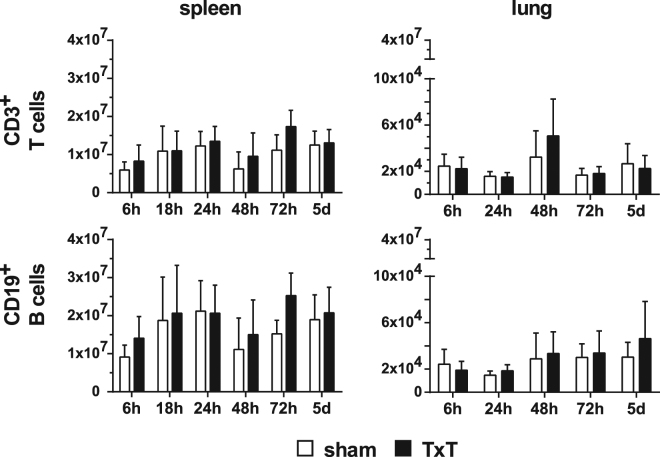
TxT does not change the numbers of lymphocytes in spleen and lung. Sham- or TxT-treated mice were analysed at different time points for the numbers of lymphocyte subsets in spleen and lung by flow cytometry. T cells were identified by staining CD3, B cell by staining CD19. Data present the mean ± SD for the following numbers of mice analysed:6 h:n = 11; 18 h:n = 8; 24 h:n = 11; 48 h:n = 11; 72 h:n = 15; 5d:n = 15. No significant statistical differences were detected between sham- and TxT-treated mice using Student’s t test by comparing cell numbers of sham and TxT-treated mice at each time point.

Next, we questioned whether blunt chest trauma also influences the numbers of lymphocytes in spleens and lungs. Mice were sham- or TxT-treated and the numbers of T-, B-, NK- and NKT cells were analysed at different time points after trauma. T- and B- cell numbers were not altered in spleen and lungs of TxT-treated mice compared to the sham-treated controls (Fig. 5). Due to the low amount of lymphocytes present in the lungs, we analysed NK- and NK- T cell numbers only in the spleen, but did not detect any differences between the treatment groups (data not shown).

### Blunt chest trauma-induced MDSCs support the production of Th1-associated cytokines

**Figure 6:**
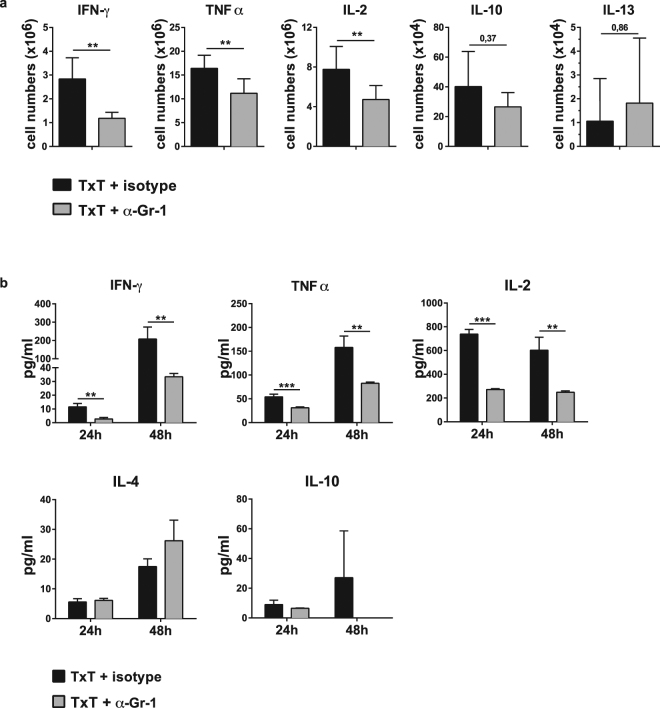
*In vivo* depletion of Gr-1^+^ cells reduces the expression of Th1-associated cytokines in TxT-treated mice. Mice were treated with the Gr-1-depleting antibody (α-Gr-1) or an isotype-specific antibody (isotype). 24 h later, TxT was induced. (**a**) 24 h after TxT, splenocytes were restimulated with PMA/Iono and the intracellular expression of Th1- and Th2-associated cytokines was determined in the CD3^+^ T cell population by flow cytometry and the number of cytokine producing T cells was calculated. Data present the mean value ± SD from 10 mice in two different experiments. (**b**) Alternatively, 24 or 48 hours after TxT, spleen cells were re-stimulated with ConA and cytokine concentrations were determined in the supernatant 24 hours after stimulation. Data show the mean value ± SD from triplicates of one mouse representative of three analysed. Significance was calculated by student’s t test comparing isotype or α-Gr-1-treated animals at each time point.

In several pathological conditions such as bone marrow transplantation, sepsis, peripheral tissue trauma or tumours^10,13,15,24^ the presence of MDSCs modulates the T cell response towards Th2 induction. To assess the polarization of T cells in the presence of TxT-derived MDSCs, mice were either injected with the Gr-1-depleting antibody anti-Gr-1 (α-Gr-1) or an isotype-specific antibody (isotype) and 24 hours later TxT was induced. After 24 hours isolated splenocytes were restimulated with PMA/ionomycin to determine the number of T cells secreting Th1 or Th2 cytokines. In the presence of Gr-1^+^ cells, most T cells produce type 1 cytokine IFN-γ and TNFα and IL-2, while only few T cells express IL-10 and -13. In the absence of Gr-1^+^ cells, the number of IFN-γ, TNFα and IL-2 producing T cells strongly declined. No change was observed in the number of IL-10 and IL-13 producing T cells (Fig. 6a). To further confirm that depletion of Gr-1 cells reduces the number of IFN-γ and TNFα producing T cells, splenocytes isolated from mice 24 or 48 hours after TxT, which were either injected with the Gr-1 depleting antibody or isotype control, were activated with ConA and cytokine expression was determined in supernatants after 24 hrs. Consistently with the decrease of IFN-γ, TNFα and IL-2 producing T cells in the absence of Gr-1^+^ cells, IFN-γ, TNFα and IL-2 concentrations in the supernatant of activated spleen cells were significantly reduced. Concentrations of Th2 cytokines IL-4 and -10 were not altered (Fig. 6b). These results clearly indicate, that the presence of Gr-1^+^ cells consisting of neutrophils and MDSCs supports the ability of T cells to induce a Th1 response and thereby activate cellular immunity.

## Discussion

Tissue injuries are characterized by severe changes in the immune response. The initial strong activation of the innate immune response is counterbalanced by a suppression of adaptive immunity favouring infectious complications, sepsis and late onset of multi organ dysfunction syndrome (MODS). Therefore, it is of major interest to determine changes in immune cells, which are involved in the regulation of the immune response. To our knowledge, we show for the first time, that blunt chest trauma induces immunosuppressive MDSCs, which prevent antigen-specific expansion of T cells and influence the Th1/Th2 balance.

MDSCs after TxT were identified by the co-expression of Gr-1 and CD11b and were induced preferentially in the lungs. The numbers increased 2–3 fold until 24 hours after TxT and no differences were detected compared to sham-treated mice at later time points. Induction of MDSCs in the spleen was hardly observed indicating that the response was prior local and not systemic. Although splenic MDSC numbers were only slightly elevated until 6 hours after TxT, MDSCs acquired an immunosuppressive phenotype. MDSCs isolated 24 hours after TxT inhibited the proliferation of alloantigen-activated CD4^+^ and CD8^+^ T cells up to nearly 80%. Thereafter the immunosuppressive capacity decreased and was not detectable after 72 hours pointing to a transient immunosuppressive status after TxT. CD11b and Gr-1, however, are markers, which are also expressed on mature myeloid cells and therefore, the inhibitory capacity to prevent T cell proliferation finally defines the existence of MDSCs. Sorting TxT-derived splenic CD11b^+^Gr-1^+^ cells by different expression levels of CD11b and Gr-1 as described by Greifenberg *et al*.^28^ showed that TxT leads to an expansion of mature neutrophils and granulocytic MDSCs. Inhibition of T cell proliferation, however, was preferentially mediated by granulocytic and monocytic MDSCs and hardly by Gr-1^high^ neutrophils. Although neutrophils are widely known as short-lived cells of the innate immune system representing the first defence line after tissue damage, it becomes more evident that they exhibit extensive plasticity regarding life time, effector functions and their role in adaptive immunity. Sagiv *et al*. discriminated between low-density neutrophils consisting of immunosuppressive immature granulocytic MDSCs with ring-shaped nuclei and immunosuppressive mature neutrophils with segmented nucleus and mature high-density neutrophils with segmented nuclei, which were not immunosuppressive^31^. Immunosuppressive neutrophils were also described in gastric cancer or cystic fibrosis patients^32,33^. Since MDSCs and neutrophils share the same markers and can both mediate immunosuppression, a clearer definition is sorely needed to define the different CD11b^+^Gr-1^+^ subpopulations.

To clarify, whether T cells induced after TxT were also inhibited in the presence of CD11b^+^Gr-1^+^ cells, the proliferative T cell response towards SEB was analysed in mice depleted for Gr-1 cells. Anti-Gr-1 antibody efficiently depleted mature neutrophils and granulocytic MDSC while the number of monocytic MDSCs was hardly reduced. TxT-induced CD4^+^ and CD8^+^ T cells exhibited decreased proliferation rates after SEB injection, if CD11b^+^Gr-1^+^ cells were present, indicating the inhibitory capacity of MDSC on T cell also *in vivo*. The gain of immunosuppressive function was accompanied by the induction of arginase-1 and iNOS, both enzymes depleting L-arginine from the environment, an amino acid essentially required for T cell proliferation. MDSC accumulation is also reported for other trauma models, while most data refer to experimental sepsis models, in which a massive increase of MDSCs after injury is observed^13,21,22,25,34^. Elevation of MDSC numbers, however, also occurs after peripheral tissue trauma, which includes soft tissue and fracture damage, and after spinal cord injury. In both models, MDSC induction is moderate, occurs shortly after injury and rapidly decreases thereafter comparable with the TxT model^23,24,26^. Independent of the model used, trauma-induced MDSCs contribute to an immunosuppressed phenotype, since they strongly impair the proliferative response of T cells activated *in vitro* ^21,24,26^. Arginase-1 expression is strongly increased in these trauma-induced MDSCs compared to MDSCs from sham-treated mice or from mice, in which MDSC expansion after trauma is pharmacologically suppressed^21,22,25^. The observed increase in iNOS expression in our TxT model was not reported in other trauma models. Probably, the particular cytokine composition induced after certain types of traumata determines the kind of immunosuppressive molecules, which are up regulated comparable to *in vitro*-generated MDSCs. In vitro-generated MDSCs are dependent on iNOS, when generated in the presence of GM-CSF, while GM-CSF/G-CSF/IL-13-induced MDSCs require arginase-1 for their immunosuppressive ability^35,36^.

The increased numbers and immunosuppressive function of MDSCs correlated with the appearance of local and systemic TxT-induced pro-inflammatory mediators such IL-6, IL-1β, and G-CSF^18^. It will be of further interest to elucidate, which trauma-induced factors favour the induction of immunosuppressive MDSCs. Endogenous glucocorticoids account for MDSC expansion in spleen, blood and bone marrow in a model of experimental sepsis, since blockade of the glucocorticoid receptor blunted the expansion of MDSCs^22^. In the same model, Li *et al*. reported that cyclooxygenase-2 strongly contributes to MDSC accumulation and the attenuation of T cell expansion^25^. On DNA levels the myeloid-differentiation-related transcription factor nuclear factor I (NFI) controls MDSC expansion and immunosuppression during sepsis^37,38^ and NFI proteins have been shown to affect the expression of genes regulated by a number of signal transduction pathways, including those controlled by TGF-β, steroid hormones, TNFα and others^39^. In a peripheral tissue trauma model the treatment with an anti-high mobility group box 1 (HMBB1) antibody ameliorates the trauma-induced attenuated T cell response and accumulation of MDSCs^24^.

MDSCs do not only interfere with T cell activation and expansion but also modulate the type of the T cell response induced. In experimental models of bone marrow and solid organ transplantation, influenza infection or sepsis, in patients with oesophageal, pancreatic or gastric cancers and in the placenta of healthy pregnant women the appearance of MDSCs is associated with the induction of Th2 cells^10–13,15,40^. Several reports suggest that tissue trauma, surgical stress or sepsis^41–43^ may shift the balance towards Th2 immune responses, which are responsible for an impaired pathogen defence and increased susceptibility to infection. Surprisingly, we observed that *in vivo* depletion of Gr-1^+^ cells during TxT did not change the number of Th2 producing T cells and their ability to produce Th2-specific cytokines. Instead, Th1-producing T cells and cytokines significantly decreased in the absence of Gr-1^+^ cells suggesting that the induction of Gr-1^high^ cells in the TxT model favour a Th1 response. Interestingly, also for a murine asthma model, representing another kind of lung inflammation, the presence of MDSCs is associated with the prevalence of Th1 cells since *in vivo* induction of MDSCs by LPS or the transfer of tumour-derived MDSCs in an asthma environment suppress Th2 effector functions^16,17^. MDSCs induction after gastrointestinal Heligmosomoides polygyrus bakeri infection is linked with a suppressed Th2 response needed for efficient nematode elimination^44^. These data strongly suggest that the type and the inflammatory status of the target organ probably influence the subtype and suppressive capacity of the developing MDSCs and subsequently the kind of T cell response initiated. While T cell responses were strongly modulated by MDSCs, the absence of CD11b^+^Gr-1^+^ cells did not influence the early local pro-inflammatory response and faintly increases the early systemic response.

Although several studies describe the influence of trauma on MDSC induction and function in mouse models, only limited data exist in the human setting. MDSCs with a granulocytic phenotype are induced in patients with sepsis. They impair T cell functions via upregulated arginase-1 and L-arginine metabolism^45–47^. High arginase levels are also detected in myeloid cells of trauma or surgery patients while plasma arginine concentrations are significantly decreased^48–50^.

Though, there is growing evidence that MDSC accumulation occurs after trauma, it is still unclear, whether the injured host profits or gains drawbacks from the presence of MDSCs. The timing of MDSC appearance and the type of trauma-induced inflammatory response might determine the maturation state, the immunosuppressive capacity and the differentiation potential of MDSCs. MDSCs isolated from septic mice exhibit a pro-inflammatory phenotype early after sepsis induction and shift to a more immature state with an anti-inflammatory profile during the progression of the disease^34^. Tumour-derived MDSCs isolated at early time points after tumour injection function as immunogenic antigen-presenting cells, while they exhibit poor immunogenicity if harvested at late time points^51^. Differentiation of tumour-derived MDSCs into macrophages and dendritic cells after adoptive transfer into healthy mice further depict the plasticity of MDSCs dependent on the inflammatory environment^52^. In the TxT model the early appearance of immunosuppressive MDSCs indicate a possible role in dampening the overwhelming trauma-induced immune response. Transferring in vitro-generated MDSCs^10^ at different time points after TxT and analysing changes in the innate and adaptive trauma-induced immune response will further clarify the immunoregulatory functions of MDSCs and indicate whether these suppressor cells might be applicable as cellular therapy to counterbalance the disequilibrated traumatic immune response.

## Methods

### Animals and blunt chest trauma (TxT)

Male C57BL/6 mice (H-2^b^, CD45.2) (Janvier, France) were used at an age between 11 to 15 weeks. Blunt chest trauma (TxT) was induced by a single blast wave centered on the thorax under sevoflurane anesthesisa as described previously^53^. In brief, compressed air was delivered in the upper section of the blast wave generator, which is divided by the lower section by a Mylar polyester film. As soon as the pressure in the upper part exceeded the resistance of the membrane, the film ruptured towards the nozzle and released a reproducible single blast wave and contusion of the lung, which is not associated with histological alterations in liver or abdomen. The sternum cylinder distance was 1.5 cm. Mice received Buprenorphin 0.03 mg/kg 30 minutes before TxT and additionally 8 and 24 hours after TxT as analgesic treatment. Sham mice were treated exactly like TxT-mice including anaesthesia and analgesic treatment with the exception that no blast wave was given. B6.SJL-Ptprc^a^Pepc^b^/BoyJ (B6.SJL; H-2^b^, CD45.1) (breeding pairs from the The Jackson Laboratory and bred at University of Ulm) and DBA/2 (H-2^d^, CD45.2) (Janvier) mice were used for T cell proliferation assays. All animal studies were carried *in accordance with* the relevant guidelines and regulations and were *approved* by the Regierungspräsidium Tübingen (1196, 1297).

### BAL fluid and blood serum

Bronchoalveolar lavage (BAL) samples were obtained as described previously^54^. Briefly, trachea was exposed, cannulated and afterwards, 0.5 ml ice-cold PBS was injected and recovered. 1 µl proteinase inhibitor cocktail was added to 100 µl BAL and samples were centrifuged at 13,000 rpm for 1 min. Supernatant was stored at −80 °C until further investigations. Blood was collected after a puncture of the mandibular vein, incubated at room temperature for 30 min and centrifuged at 13,000 rpm at 4 °C for 15 min. Cytokine stabilization buffer (U-CyTech biosciences) was added to the serum and stored at −80 °C until cytokine analysis by Procartaplex Multiplex Immunoassays (Thermo Fisher Scientific).

### ***In vivo*** depletion of MDSCs

Mice were injected with 250 µg anti-Gr-1 antibody (anti mouse Ly6G/Ly6C, cl. RB6-8C5, BioXCell) or 250 µg isotype control rat-IgG2b (cl. LTF-2, BioXCell) i.p. in 200 µl PBS one day before TxT. In SEB experiments injection of α-Gr-1 or isotype was repeated 48 hrs after the 1^st^ injection. Depletion efficiency was controlled by staining cells for CD11b and Gr-1 and ranged between 80–90%.

### Staphylococcus enterotoxin B (SEB) treatment

α-Gr-1- or isotype control- (rat-IgG2b) treated mice underwent TxT and 24 hours after TxT 50 µg SEB (Sigma-Aldrich)/mouse or PBS was injected iv in the tail vein. Expansion of vß8^+^ T cells was determined 48 h later by flow cytometry.

### Magnetic bead isolation

CD11b^+^ cells from the spleen were positively selected according to manufacture’s protocol using CD11b MicroBead Kit (Milteny). Selected cells were afterwards stained for the co-expression of Gr-1. Purity of isolated cells ranged between 75–85%.

### Flow cytometry

A total of 5 × 10^5^ cells were stained in FACS-medium (PBS − 10% FCS − 0.2%NaN_3_) with the following antibodies Gr-1 (cl. RB6–8C5), CD11b (cl. M1/70), CD11c (cl. N418), CD3 (cl. 17A2), CD4 (cl. RM4-5), CD8 (cl. 53-6.7), CD19 (cl. 1D3), Ly-6C (cl. HK1.4) (Thermo Fisher Scientific), CD45.1 (cl. A20), vβ8.1.2.3 (cl. F23.1), Ly-6G (cl. 1A8) (BD Bioscience). Dead cells were gated out by analysing 7-amino-actinomycin-D (7-AAD, Sigma-Aldrich) negative cells. Flow cytometry samples were measured on LSR II flow cytometer (BD Bioscience). To sort CD11b^+^Gr-1^+^ cell subpopulations from spleen cells, B cells were depleted according to manufacture’s protocol using BioMag goat anti-mouse IgG (Qiagen), remaining cells were stained with CD11b and Gr-1 and afterwards sorted with FACSAria^TM^III flow cytometer (BD Bioscience).

### CFSE labelling

2 × 10^6^ spleen cells were labelled with 5 µM CFSE (Thermo Fisher Scientific) at 37 °C for 10 min, immediately washed with ice-cold PBS-5%FCS, and subsequently used for proliferation assays.

### Mixed lymphocyte reaction (MLR)

To determine the immunosuppressive function of TxT-induced MDSCs, MDSCs were isolated from TxT- or sham-treated mice as described at different time points after trauma. 2.5 × 10^6^/ml CFSE-labelled effector spleen cells (B6.SJL) were activated with the same number of allogeneic irradiated spleen cells (DBA/2) in the presence of decreasing MDSC numbers and proliferation was determined on day 4 by flow cytometry. Percentage suppression = 100 − (% proliferating T cells + stimulators + MDSCs − % proliferation of T cells in medium alone)/(% proliferating T cells + stimulators − % proliferation of T cells in medium alone) × 100.

### Cytokine expression

Cytokine expression was determined intracellular by activation of spleen cells with PMA (20 ng/ml) plus ionomycin (1 µM) (Calbiochem) in the presence of Brefeldin A (10 µg/ml; Sigma-Aldrich). After 5 hours, cells were stained for CD3, fixed with 4% paraformaldehyde, subsequently lysed with 0.1% saponin (Sigma-Aldrich), and stained for the different cytokines. The following cytokine-specific antibodies were used: IFN-γ (cl. XMG1.2), IL-2 (cl. JES6-5H4), IL-10 (cl. JES5-16E3), IL-13 (cl. eBio13A) (Thermo Fisher Scientific), TNFα (cl. MP6-XT22) (BioLegend). Alternatively, cytokine secretion was analysed by activating splenocytes in the presence of 2.5 µg/ml Con A (Sigma-Aldrich). 24 hours later, cytokine concentrations in the supernatants were defined by Procartaplex Multiplex Immunoassays (Thermo Fisher Scientific) and analysed on a BIO RAD-Bio-Plex 200 System (Bio-Rad).

### RNA preparation and quantitative reverse-transcription polymerase chain reaction (qRT-PCR)

RNA was isolated using the Direct-zol™ RNA Mini Prep Kit (Zymo Research, Freiburg) and complementary DNA was synthesized using SuperScript Reverse Transcriptase (Life Technologies). qRT-PCR was performed with a Light Cycler 2.0 using a LightCyler FastStart DNA Master PLUS SYBR Green I Kit (Roche Diagnostics). The qRT-PCR results were normalized using mouse aryl hydrocarbon receptor-interacting protein (AIP) as house keeping gene. The following primers (Thermo Fisher Scientific) were used: AIP (forward: 5′-GCTCCGTTATAGATGACAGC-3′, reverse: 5′-ATCTCGATGTGGAAGATGAG-3′), arginase 1 (forward: 5′-TCCTTTCAAATTGTGAAGAACCCACGGTC-3′, reverse: 5′-AGAATCCTGGTACATCTGGGAACTTTCCT-3′), iNOS (forward: 5′-AGCAATGGGCAGACTCTGAAGAAATCTC-3′, reverse: 5′-ATGTTTGCTTCGGACATCAAAGGTCTCAC-3′).

### Statistics

Data were analysed by either using a Student’s t or one-way ANOVA followed by a Sidak test as a post hoc test for multiple comparisons. Results were considered as significant if P ≤ 0.05.

### Data availability

The data sets generated during the current study are available from the corresponding author on reasonable request.

## Electronic supplementary material

Supplementary Information
