# Supplementary material for: MDSCs are induced after experimental blunt chest trauma and subsequently alter antigen-specific T cell responses
Source: Sci Rep. 2017 Oct 9;7:12808. doi: 10.1038/s41598-017-13019-6 (PMC5634472; doi:10.1038/s41598-017-13019-6)
Supplement: Supplementary file 1 — Supplementary Information [file 41598_2017_13019_MOESM1_ESM.pdf]

**MDSCs are induced after experimental blunt chest trauma and subsequently alter antigen-specific T cell responses**

Yvonne Hüsecken[1], Sylvia Muche[1], Monika Kustermann[1], Malena Klingspor[1], Annette Palmer[2], Sonja Braumüller[2], Markus Huber-Lang[2], Klaus-Michael Debatin[1] and Gudrun Strauss[1*]

1 Department of Pediatric and Adolescent Medicine, University Medical Center Ulm, Germany

2 Institute of clinical and Experimental Trauma Immunology, University Medical Center Ulm, Germany

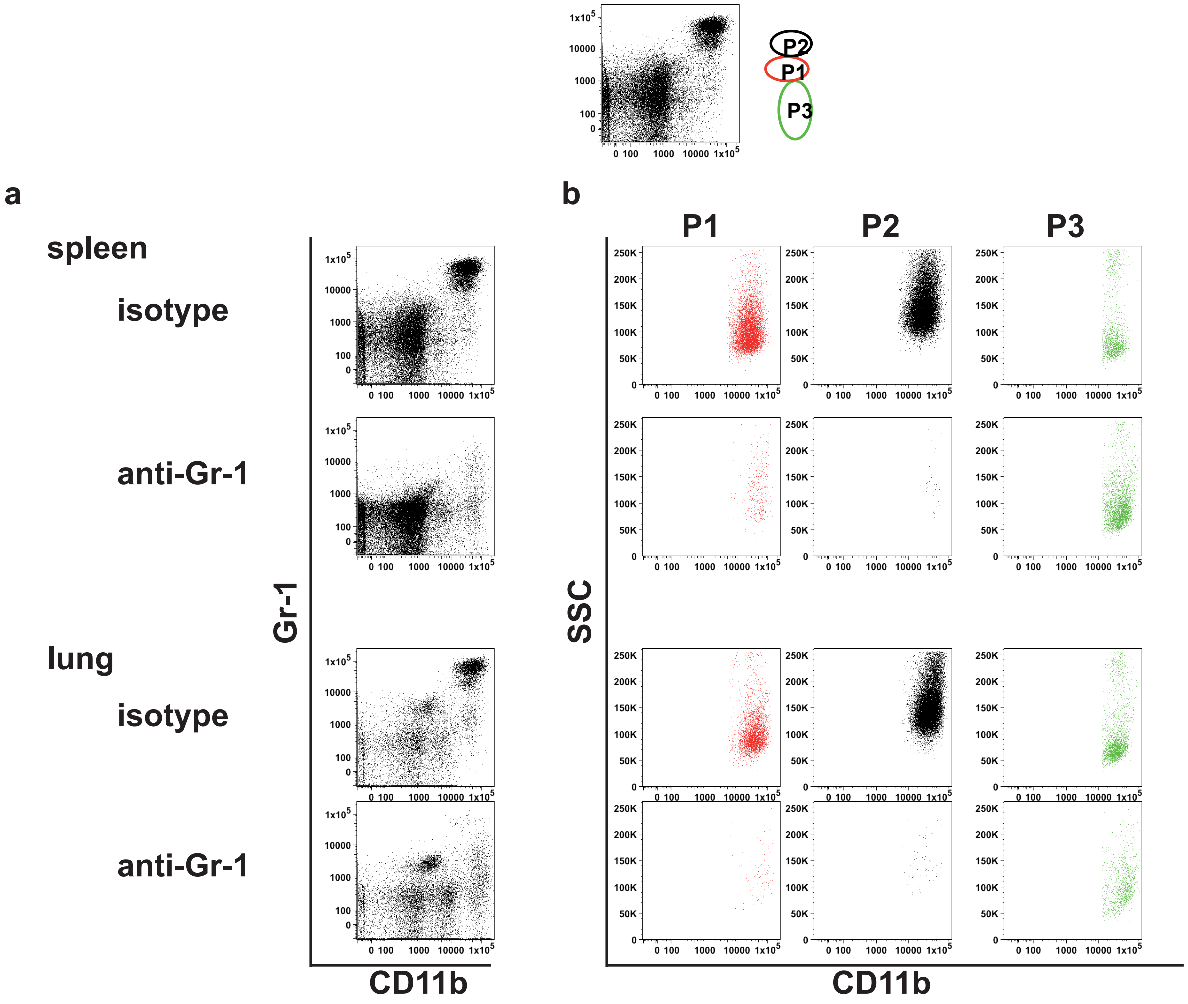


**Supplementary Figure S1: Anti-Gr-1 treatment efficiently depletes Gr-1$^{high}$CD11b$^{+}$ cells**
Mice were injected either with anti-Gr-1 antibody or isotype control 24 hours before TxT. a) 24 hours after Txt, spleen and lung cells were stained with anti-Gr-1 and CD11b antibodies. b) To further prove $\alpha$-Gr-1-mediated cell depletion, Gr-1 positive cells were gated (P1, P2, P3) on cells of the isotype-treated animals and gates were copied on cells of anti-Gr-1 treated animals. Cells in P1, P2 and P3 were depicted as CD11b/SSC flow diagrams.

# Supplementary Figure S1

**MDSCs are induced after experimental blunt chest trauma and subsequently alter antigen-specific T cell responses**

Yvonne Hüsecken[1], Sylvia Muche[1], Monika Kustermann[1], Malena Klingspor[1], Annette Palmer[2], Sonja Braumüller[2], Markus Huber-Lang[2], Klaus-Michael Debatin[1] and Gudrun Strauss[1*]
1 Department of Pediatric and Adolescent Medicine, University Medical Center Ulm, Germany
2 Institute of clinical and Experimental Trauma Immunology, University Medical Center Ulm, Germany

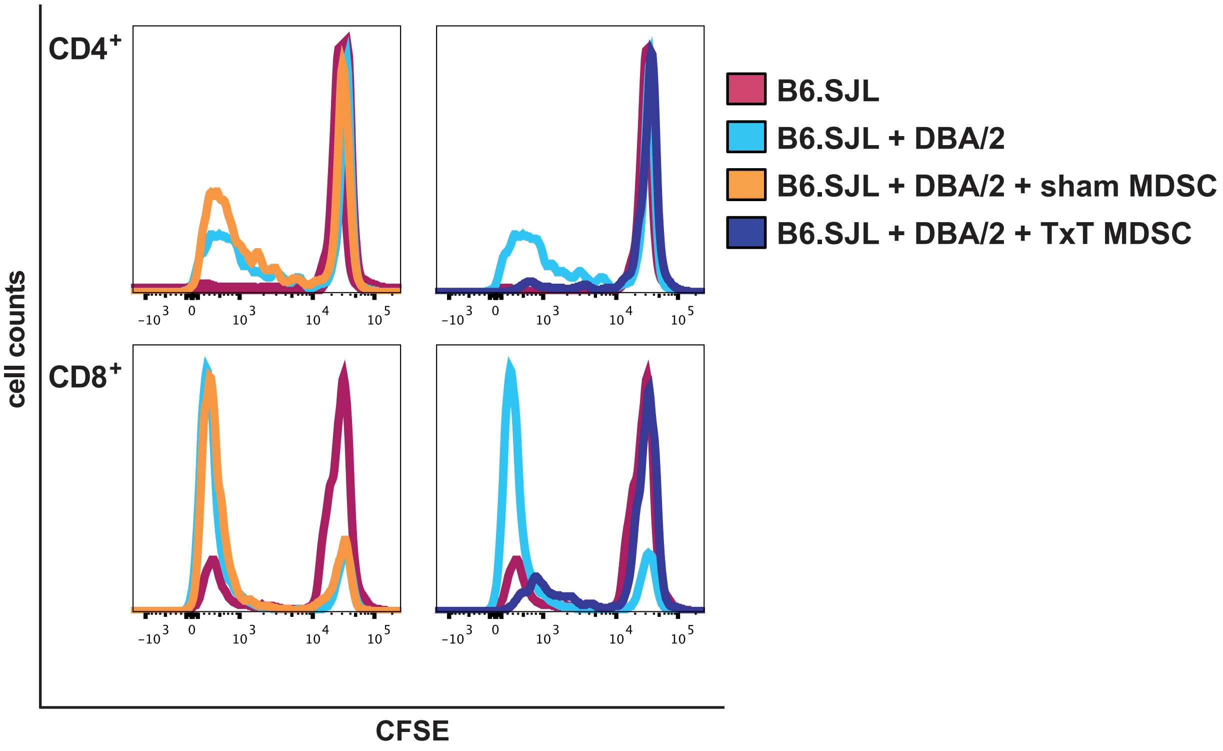


**Supplementary Figure S2: Representative flow data for MDSC-mediated inhibition of alloantigen-specific T cell proliferation**

MDSCs were isolated by magnetic beads from spleens at 24 hours from sham- or TxT-treated mice and co-cultivated with CFSE-labeled spleen cells of B6.SJL mice (H-$2^b$, CD45.$1^+$), which were activated alloantigen-specifically with irradiated spleen cells of DBA/2 mice (H-$2^d$, CD45.$2^+$). MDSCs were added with a MDSC:T cell ratio of 1:1. After 4 days, cells were stained for CD45.1, CD4, CD8 and 7-AAD and proliferation of living CD45.$1^+$CD$4^+$ and CD45.$1^+$CD$8^+$ T cells was analyzed by CFSE dilution.

## Supplementary Figure S2
